# A New Pharmacogenetic Algorithm to Predict the Most Appropriate Dosage of Acenocoumarol for Stable Anticoagulation in a Mixed Spanish Population

**DOI:** 10.1371/journal.pone.0150456

**Published:** 2016-03-15

**Authors:** Hoi Y. Tong, Cristina Lucía Dávila-Fajardo, Alberto M. Borobia, Luis Javier Martínez-González, Rubin Lubomirov, Laura María Perea León, María J. Blanco Bañares, Xando Díaz-Villamarín, Carmen Fernández-Capitán, José Cabeza Barrera, Antonio J. Carcas, PGX-ACE Investigators Group

**Affiliations:** 1 Department of Clinical Pharmacology, La Paz University Hospital, IdiPAZ, Madrid, Spain; 2 Department of Clinical Pharmacy, San Cecilio University Hospital, Institute for Biomedical Research, Ibs, Granada, Spain; 3 Genomics Unit, Centre for Genomics and Oncological Research (GENYO), Pfizer-University of Granada-Andalusian Regional Government, Health Sciences Technology Park, PTS, Granada, Spain; 4 Department of Pharmacology, School of Medicine, Autonomous University of Madrid, IdiPAZ, Madrid, Spain; 5 Department of Hematology, La Paz University Hospital, Madrid, Spain; 6 Department of Internal Medicine, La Paz University Hospital, IdiPAZ, Madrid, Spain; National Cancer Center, JAPAN

## Abstract

There is a strong association between genetic polymorphisms and the acenocoumarol dosage requirements. Genotyping the polymorphisms involved in the pharmacokinetics and pharmacodynamics of acenocoumarol before starting anticoagulant therapy would result in a better quality of life and a more efficient use of healthcare resources. The objective of this study is to develop a new algorithm that includes clinical and genetic variables to predict the most appropriate acenocoumarol dosage for stable anticoagulation in a wide range of patients. We recruited 685 patients from 2 Spanish hospitals and 1 primary healthcare center. We randomly chose 80% of the patients (n = 556), considering an equitable distribution of genotypes to form the generation cohort. The remaining 20% (n = 129) formed the validation cohort. Multiple linear regression was used to generate the algorithm using the acenocoumarol stable dosage as the dependent variable and the clinical and genotypic variables as the independent variables. The variables included in the algorithm were age, weight, amiodarone use, enzyme inducer status, international normalized ratio target range and the presence of CYP2C9*2 (rs1799853), CYP2C9*3 (rs1057910), *VKORC1* (rs9923231) and *CYP4F2* (rs2108622). The coefficient of determination (R^2^) explained by the algorithm was 52.8% in the generation cohort and 64% in the validation cohort. The following R^2^ values were evaluated by pathology: atrial fibrillation, 57.4%; valve replacement, 56.3%; and venous thromboembolic disease, 51.5%. When the patients were classified into 3 dosage groups according to the stable dosage (<11 mg/week, 11–21 mg/week, >21 mg/week), the percentage of correctly classified patients was higher in the intermediate group, whereas differences between pharmacogenetic and clinical algorithms increased in the extreme dosage groups. Our algorithm could improve acenocoumarol dosage selection for patients who will begin treatment with this drug, especially in extreme-dosage patients. The predictability of the pharmacogenetic algorithm did not vary significantly between diseases.

## Introduction

Despite the development of new oral anticoagulants, coumarins are still the most widely used anticoagulants for treating and preventing thromboembolism. Warfarin is the most frequently prescribed coumarin worldwide, although acenocoumarol and phenprocoumon are preferentially used in some countries. Although these vitamin K antagonists are effective for preventing cardioembolic stroke, myocardial infarction and venous thrombosis, they double the incidence of hemorrhage, and this risk is particularly high during the first month of therapy [1]. Accurate dosing of coumarin anticoagulants is challenging due to the wide interindividual and intraindividual variability in the dosage necessary to achieve stable anticoagulation.

In the last decade, the identification of genetic variants influencing the metabolism of coumarins led to the publication of numerous articles focused on the potential of pharmacogenetic information to personalize therapy and to anticipate the best therapeutic dosage for patients starting coumarin treatment. Thus, the use of dosing algorithms that include genetic and nongenetic factors has been the most common strategy for predicting the most appropriate dosage of antivitamin-K oral anticoagulants [2–7].

A wide variety of studies, including several genome-wide association studies (GWAS), have identified the primary genetic variants that influence warfarin and acenocoumarol dosing [8–14]. Patients with variant alleles of *CYP2C9*, the primary enzyme that metabolizes S-warfarin and S-acenocoumarol, require reduced dosages compared with those having wild-type alleles. Warfarin and acenocoumarol dosing variability is also attributable to genetic polymorphisms in vitamin K 2,3 epoxide reductase complex 1 (*VKORC1*). In addition, a subsequent finding revealed that *CYP4F2* genetic variants were associated with a clinically relevant effect on warfarin requirements in the Caucasian population [13].

To obtain personalized warfarin dosages, several models have been developed that include these genetic variants and demographic and clinical factors for various patient populations. These algorithms explain approximately 51–59% of the variation in warfarin doses [4,15–17]. Although warfarin and acenocoumarol are very similar, they differ in their pharmacokinetic and pharmacodynamic characteristics and their genetic influence. Warfarin algorithms cannot therefore be applied to acenocoumarol [18].

To date, a number of algorithms have also been published for acenocoumarol in diverse populations. Verde et al. constructed an ‘‘acenocoumarol-dose genotype score” based on the number of alleles associated with a higher acenocoumarol dosage taken by each participant for each polymorphism [19]. Two algorithms have been published for Indian populations, which include demographic, clinical and genetic variants, and have obtained coefficients of determinations of 41% and 61.5%, respectively [20,21]. In addition, 3 other algorithms have been developed for European populations. The first algorithm, designed for a mixed population, was published by the European Pharmacogenetics of Anticoagulant Therapy (EU-PACT) study group and includes *CYP2C9* and *VKORC1* polymorphisms and clinical variables (age, sex, weight, height and amiodarone use). When applied to the EU-PACT cohort, this algorithm explained 52.6% of the dosage variance, whereas the nongenotype algorithm explained 23.7% [22]. Another algorithm was developed by our group (La Paz University Hospital, LPUH) for a cohort of patients with Thromboembolic Venous Disease (TVD) and considers the influence of clinical variables (age, body mass index [BMI], amiodarone use and enzyme-inducer use) and genetic variations of *CYP2C9*, *VKORC1*, *CYP4F2* and *APOE*. For our entire cohort, this algorithm predicted 56.8% of the dose variance, whereas the clinical factors explained only 19% of the variability [23]. The last algorithm was developed by Cerezo-Manchado et al. in a large cohort of patients undergoing anticoagulation therapy (n = 973) and includes clinical factors (age and BMI) and genetic variants (*VKORC1*, *CYP2C9* and *CYP4F2* polymorphisms). The algorithm explained 50% of the variance in the acenocoumarol dosage, whereas the clinical algorithm explained 16% [24].

The published algorithms differ in the variables included and the variables’ weight and show differences in the population and methods used to develop the predictive models. The clinical variables included in the algorithms differ essentially in terms of the inclusion or not of sex or in terms of using weight and height instead of BMI. In addition, a number of algorithms take into account the use of amiodarone and enzyme-inducer drugs while others do not. In terms of the genetic variants, all algorithms included *CYP2C9* and *VKORC1* polymorphisms. *CYP4F2* is taken into account in a number of algorithms, and *APOE* is used only by the LPUH group.

LPUH algorithm [23] was developed for a well-defined prospective cohort of patients with TVD [Deep Vein Thrombosis (DVT) and Pulmonary Embolism (PE)], whereas the other algorithms [22,24] have typically included patient cohorts with various anticoagulation indications (valve replacement [VR], atrial fibrillation [AF] and TVD). The objective of this study is to develop a new algorithm in a larger prospective cohort of patients that would be useful for a wider range of patients.

## Materials and Methods

### Study design and patients

Written informed consent was obtained from all patients prior to their participation in the study. Ethics approval for this study was obtained from both clinical research ethics committees: LPUH of Madrid and San Cecilio University Hospital (SCUH) of Granada, Spain. This was an observational, cross-sectional study. Patients were recruited from the various clinical departments of LPUH of Madrid and SCUH of Granada and a primary health care center (PHCC) belonging to LPUH. Participants included 685 patients who were treated according to the disease in the various departments of the 2 hospitals and the PHCC. Inclusion criteria included acenocoumarol anticoagulation for AF or TVD with a target INR of 2–3, and VR or other diseases (OD) requiring anticoagulation, with a target INR of 2.5–3.5. The participants were taking a stable dosage of acenocoumarol, defined as a weekly dose variation of <20% in the last 3 consecutive months and an INR within 2–3 (AF and TVD) and 2.5–3.5 (VR and OD) for at least the last 3 consecutive months. Exclusion criteria included renal (estimated creatinine clearance ≤30 mL/min) and hepatic (Child-Plough stage) impairment, thyroid dysfunction and cancer.

The INR was measured according to the technique used in each recruiting center; all centers have quality certifications with external controls. In LPUH the analysis was performed using a semiautomated Thrombotrack® CoaguLometer (Baxter) with Thrombotest reagents. SCUH used the microINR from iLine microsystems, and PHCC used a portable INRatio (Grifols) coagulometer.

### Data collection and genotyping

The collected data included age, sex, race, body weight and height, INR results, acenocoumarol dose administered in the last 3 consecutive months, concomitant medications and target INR range. Blood samples were collected in tubes containing ethylenediaminetetraacetic acid (LPUH) and samples of buccal mucosa cells (SCUH) and stored at -20°C. For DNA extraction, the QuickGene DNA blood kit S (Fujifilm, Düsseldorf, Germany) was used in LPUH; the DNA extraction procedure in SCUH was performed according to the method previously published by Freeman et al. [25], with a number of modifications described by Gómez-Martín A. et al. [26]. KASPar® (KBiosciences, Hoddesdon, UK), and TaqMan® (Applied Biosystems, Foster City, USA) technologies were used for genotyping. The genotype frequencies were calculated and the distributions tested for Hardy-Weinberg equilibrium.

We randomly chose 80% of the patients (n = 556), considering an equitable genotype distribution (*CYP2C9*, *VKORC1* and *CYP4F2*) to form the generation cohort. The remaining 20% (n = 129) formed the validation cohort of the new algorithm.

### Statistical analysis

The results for the categorical data were expressed in absolute terms; such as percentages and the comparison between groups were performed using the chi-squared test. The continuous variables were expressed as means and standard deviation (SD) and were compared using Student´s t-test and ANOVA.

Univariate analyses were performed for each variable (statistically significant P≤.05); however, those reaching p-values less than 0.1 were included in a multivariate analysis. We ultimately chose multiple linear regression by the enter method to generate the algorithm, given the method would be more easily transformable into a useful equation in routine clinical practice. After this process, we selected those variables with p-values consistently below .05 to be included in the pharmacogenetic algorithm. In the case of variables near significant with biological plausibility and previous evidence of association with acenocoumarol dosing, were also included in the algorithm. The dependent variable was the acenocoumarol dosage required (ln-transformed) to obtain a stable target INR range. As independent variables, we included the selected demographic, clinical and genetic variables. To elucidate the contribution of the patients´ demographic and clinical characteristics as currently used in clinical practice, a clinical algorithm was designed including only those variables used in the pharmacogenetic algorithm.

The performance of pharmacogenetic and clinical algorithms was evaluated by calculating the coefficient of determination (R^2^) that represents the variability explained by each model. The accuracy and precision of the model were assessed using the mean error (ME; mean of the differences between the predicted and observed acenocoumarol dosages) and the mean absolute error (MAE; mean absolute difference between the predicted and observed acenocoumarol dosages). The ME and MAE were also calculated as the percentages of the observed acenocoumarol dosage (%ME and %MAE). The ME reflects the accuracy of the prediction, and MAE is an estimate of the model´s precision.

### Clinical relevance

To evaluate the clinical relevance of the model, we classified the patients into 3 groups according to the actual dosage administered: a low-dosage group (<11 mg/week), an intermediate-dosage group (11–21 mg/week) and a high-dosage group (>21 mg/week). We calculated the proportion of patients for whom the predicted dosage was within 20% of the actual dosage (considered correctly dosed). We evaluated the predicted dosage by applying the pharmacogenetic and clinical algorithms.

All analyses were performed using SPSS 16.0 (IBM Inc., IL, USA).

## Results

### Demographics

The generation cohort consisted of 556 patients; 283 of whom were women (50.9%); the mean (SD) age was 68.7 (0.53) years. The validation cohort consisted of 129 patients, with a mean age of 67.6 (1.3) years, 54 of whom were women (41.9%). There were missing data in both groups: amiodarone use for 2 patients in the generation cohort and the weight and height in 1 patient in the validation cohort. Excluding the patients with missing data, 554 patients comprised the generation cohort, and 128 patients comprised the validation cohort. The clinical and demographic features and genotype frequency of both groups are shown in **Table 1**. The most common disease in our complete cohort was AF (47%) followed by TVD (29.5%). When comparing the patients’ features between the various diseases (**Table 2)**, we found that patients with AF were older (72.44 ± 0.5 years) and had higher BMI (29.55 ± 0.27) (p < .001), and the acenocoumarol dosage (13.49 ± 0.33 mg/week) was significantly lower than that of the other groups (p < .001). Regarding the concomitant medication, this group included more patients treated with amiodarone (9%) compared with the groups with other diseases (p < .001). There were no differences in the genotype distribution between the various diseases.

**Table 1:** Patients characteristics in the generation (n = 556) and validation (n = 129) cohorts.

Variables	Generation cohort (n = 556)	Validation cohort (n = 129)	p
Sex, n (%)			
Female/Male	283/273 (50.9/49.1)	54/75 (41.9/58.1)	.078
Mean age, y (SD)	68.7 (12.41)	67.6 (14.80)	.446
Mean weight, kg (SD)	75.8 (14.16)	78.06 (16.26)◆	.147
Mean height, m (SD)	1.62 (0.09)	1.64 (0.10)◆	.107
Mean body mass index, kg/m2 (SD)	28.73 (4.86)	28.93 (4.30)◆	.653
Underlying disease, n (%)			
Thromboembolic venous disease	160 (28.8)	42 (32.6)	
Auricular fibrillation	263 (47.3)	59 (45.7)	
Valve replacement	115 (20.8)	26 (20.2)	
Other diseases	18 (3.2)	2 (1.6)	
Mean acenocoumarol dosage, mg/week (SD)	15.16 (0.30)	15.5 (0.67)	.632
Concurrent medications			
Inductor drugs* [Yes/No], n (%)	107/448 (19.3/80.7)γ	29/100 (22.5/77.5)	.412
Amiodarone [Yes/No], n (%)	27/527 (4.9/94.6)¥	9/120 (7/93)	.380
Phenotype, n (%)			
≤11 mg/week	177 (31,8)	39 (30.2)	
11–21 mg/week	279 (50.2)	62 (48.1)	
≥21 mg/week	100 (18)	28 (21.7)	
*CYP2C9* genotype, n (%)			.881
*1/*1	325 (58.5)	77 (59.7)	
*1/*2	138 (24.8)	30 (23.3)	
*1/*3	62 (11.2)	16(12.4)	
*2/*2	12 (2.2)	2 (1.6)	
*2/*3	14 (2.5)	4 (3.1)	
*3/*3	5 (0.9)	0 (0)	
*VKORC1* genotype, n (%)			.874
Homozygote wt/wt	202 (36.4)	47 (36.4)	
Heterozygote	277 (49.8)	62 (48.1)	
Homozygote mut/mut	77 (13.8)	20 (15.5)	
*CYP4F2* genotype, n (%)			
MM	83 (14.6)	14 (12.3)	.455
*APOE* rs7412 genotype, n (%)			.621
Homozygote wt/wt	494 (88.9)	116 (89.9)	
Heterozygote	58 (10.4)	13 (10.1)	
Homozygote mut/mut	4 (0.7)	0 (0)	

Abbreviations: SD, standard deviation; mut, mutated; wt, wild type.

* CYP inducers that were considered in this analysis included phenytoin, carbamazepine and rifampin

γ Missing data, n = 555

¥ Missing data, n = 554

◆ Missing data, n = 128

**Table 2:** Patients characteristics according to disease in the entire cohort.

Variables	TVD (n = 202)	AF (n = 322)	VR (n = 141)	OD (n = 20)	p
Sex, n (%)					
Men	100 (49.5)	176 (54.7)	63 (44.7)	9 (45)	.218
Women	102 (50.5)	146 (45.3)	78 (55.3)	11 (55)	
Mean age, y (SD)	66.12 (16.91)	72.44 (8.90)*	64.37 (10.32)	57.65 (15.83)	< .001
Mean weight, kg (SD)	76.51 (16.20)	78.43 (13.44) ¥	70.93 (13.84)	76.15 (10.84)	< .001
Mean height, m (SD)	1.63 (0.091)	1.63 (0.091) ¥	1.61 (0.10)	1.63 (0.083)	.302
Mean body mass index, kg/m2 (SD)	28.67 (5.08)	29.55 (4.77) ¥	27.15 (3.77)	28.81 (4.46)	< .001
Median acenocoumarol dosage, mg/week (range)	15 (2.5–47)	13.49 (3.0–37)*	16.48 (2.0–38.5)	21 (10.0–61.5)	< .001
Concurrent medications, n (%)					
Enzyme inducers †	25 (12.5)	80 (24.8)	26 (18.4)	5 (25)	.006
Amiodarone	2 (1)	29 (9)* ¥	5 (3.5)	0	< .001
Phenotype, n (%)					< .001
<11 mg/week	66 (32.7)	117 (36.3)	31 (22)	2 (10)	
11–21 mg/week	86 (42.6)	169 (52.5)	75 (53.2)	11 (55)	
>21 mg/week	50 (24.8)	36 (11.2)	35 (24.8)	7 (35)	
CYP2C9 genotype, n (%)					.084
Homozygote wt/wt	109 (54)	187 (58.1)	93 (66)	13 (65)	
Heterozygote	84 (41.58)	112 (34.8)	45 (31.9)	5 (25)	
Homozygote mut/mut	33 (16.3)	43 (13.4)	19 (13.5)	2 (10)	
CYP4F2 genotype, n (%)					
MM	26 (12.9)	53 (16.5)	16 (11.3)	2 (10)	.411
APOE rs7412 genotype, n (%)					.311
Homozygote wt/wt	176 (87.13)	292 (90.7)	127 (90.1)	15 (75)	
Heterozygote	24 (11.9)	29 (9)	13 (9.2)	5 (25)	
Homozygote mut/mut	2 (1)	1 (0.3)	1 (0.7)	0	

Abbreviations: AF, atrial fibrillation; OD, other diseases; SD; standard deviation; VR, valve replacement; TVD, thromboembolism venous disease; mut, mutated; wt, wild type.

† CYP inducers that were considered in this analysis included phenytoin, carbamazepine and rifampin

* Group with significant differences compared with other groups

¶ Missing data, n = 201

¥ Missing data, n = 321

### Acenocoumarol dose algorithm

The clinical variables that were ultimately included in the algorithm were age (y), weight (kg), amiodarone use, enzyme inducer status and INR target range (2–3 or 2.5–3.5). The genetic variables that remained significant in the algorithm were CYP2C9*1/*2, CYP2C9*1/*3, *CYP2C9* homozygous variants, *VKORC1* heterozygous, *VKORC1* homozygous variant and the *CYP4F2* homozygous variant. The outcome was the natural logarithm of the mean weekly doses of acenocoumarol **(Table 3)**. We grouped the polymorphisms of the *CYP2C9* homozygous variants because their function appears to be similar. The *APOE* genotype was not included in the final model because it did not reach statistical significance (p = .521, R^2^ = 0.1%).

**Table 3:** Pharmacogenetic and clinical algorithms.

**Pharmacogenetic algorithm**				
Beta	Variable	P-value	Adj R^2^ (%)	Cumulative R^2^ (%)
3.181	Intercept			
-0.010	Age	< .001		
0.005	Weight	< .001		
0.070	Enzyme inducer status	.053		
-0.337	Amiodarone status	< .001		
0.086	INR target range	.014		
Clinical variables			13.1	13.1
-0.111	CYP2C9 *1/*2	< .001		
-0.323	CYP2C9 *1/*3	< .0001		
-0.691	CYP2C9 *2/*2 or *2/*3 or *3/*3	< .0001		
*CYP2C9*			14.3	27.4
-0.302	*VKORC1 A/G*	< .001		
-0.727	*VKORC1 A/A*	< .001		
*VKORC1*			22.9	50.3
0.214	*CYP4F2 MM*	< .001		
*CYP4F2*			2.5	52.8
**Clinical Algorithm**				
2.951	Intercept			
-0.011	Age	< .001		
0.004	Weight	.013		
0.045	Enzyme inducer status	.357		
-0.290	Amiodarone status	< .001		
0.086	INR target range	.014		
Clinical variables			13.1	13.1

Beta: standardized regression coefficient

The pharmacogenetic algorithm: natural logarithm of the mean weekly doses of acenocoumarol = 3.181–0.010*age (y) + 0.005*weight (kg) + 0.070 (if enzyme inducer is used)– 0.337 (if amiodarone is used)– 0.111 (if CYP2C9*1/*2)– 0.323 (if CYP2C9*1/*3)– 0.691 (if *CYP2C9* homozygote mutated)– 0.302 (if *VKORC1 A/G*)– 0.727 (if *VKORC1 A/A*) + 0.214 (if *CYP4F2 MM*) + 0.086 (if INR target is 2.5–3.5). The outcome is the natural logarithm of the mean acenocoumarol dosage in mg/week.

### Variability of the pharmacogenetic algorithm

The variability explained by the pharmacogenetic algorithm was 52.8% in the generation cohort and increased to 64%, in the validation cohort, whereas the clinical algorithm explained 13.1% and 21.1% respectively. The accuracy of the pharmacogenetic algorithm in both cohorts was good, with a low calculated ME (-0.11 mg for the generation cohort and 0.04 mg for the validation cohort), whereas the accuracy for the clinical algorithm was higher: -1.55 (6.57) and -1.62 (6.41), respectively. The precision calculated as MAE was good in the pharmacogenetic algorithm: the MAE was 3.77 mg (3.48) in the generation cohort and 3.54 mg (2.99) in the validation cohort. For the clinical algorithm, the MAE was higher 4.99 (4.55) and 5.04 (4.25), respectively. We also calculated the predicted dosage by both algorithms in the entire cohort. For this cohort, the R^2^ was 15.1% for the clinical algorithm and 55% for the pharmacogenetic algorithm. The ME and MAE were lower in the pharmacogenetic algorithm, which means that the bias and precision were clearly better in this algorithm **(Table 4)**.

**Table 4:** Predictive performance of the pharmacogenetic and clinical algorithms in the generation, validation and entire cohorts.

	Pharmacogenetic Algorithm			Clinical Algorithm		
	Generation Cohort	Validation Cohort	Entire Cohort	Generation Cohort	Validation Cohort	Entire Cohort
**R2**	52.8%	64%	55%	13.1%	21.1%	15.1%
**ME**	-0.11 (3.48)	0.04 (4.65)	-0.09 (5.04)	-1.55 (6.57)	-1.62 (6.41)	-1.56 (6.54)
**MAE**	3.77 (3.48)	3.54 (2.99)	3.73 (3.39)	4.99 (4.55)	5.04 (4.25)	4.99 (4.49)
**%ME**	10.15 (38.17)	9.95 (35.85)	10.12 (37.72)	9.62 (62.76)	8.95 (54.9)	9.49 (61.32)
**%MAE**	28.52 (27.3)	26.64 (25.89)	28.17 (27.03)	38.97 (50.1)	38.21 (40.29)	38.83 (48.38)

Abbreviations: ME: mean error (predicted–observed); %ME: mean error expressed as a percentage (%ME = ME/Observed*100); MAE: mean absolute error (= SQR[(Predicted-Observed)^2^]); %MAE: mean absolute error expressed as a percentage (%MAE = MAE/Observed*100).

The predictive performance of the pharmacogenetic algorithm was evaluated by disease. As shown in **Table 5**, the patient group with the best prediction was the AF group, with an R^2^ of 57.4%, followed by the VR group, with an R^2^ of 56.3%.

**Table 5:** Predictive performance of the pharmacogenetic algorithm by disease in the entire cohort (n = 682).

	TVD (n = 202)	AF (n = 322)	VR (n = 141)	OD (n = 20)
**R2**	51.5%	57.4%	56.3%	45.2%
**ME**	-1.12 (5.53)	0.59 (4.07)	0.22 (5.16)	-2.59 (9.43)
**MAE**	4.12 (3.85)	3.28 (2.47)	3.91 (3.36)	5.81 (7.78)
**%ME**	2.11 (37.19)	15.23 (38.07)	11.47 (36.52)	-1.19 (31.80)
**%MAE**	26.55 (26.06)	29.68 (28.26)	27.32 (26.73)	26.13 (17.15)

Abbreviations: AF, atrial fibrillation; OD, other diseases; VR, valve replacement; TVD, thromboembolism venous; ME: mean error (predicted–observed); %ME: mean error expressed as a percentage (%ME = ME/Observed*100); MAE: mean absolute error (= SQR[(Predicted-Observed)^2^]); %MAE: mean absolute error expressed as a percentage (%MAE = MAE/Observed*100).

### Clinical Relevance

To test the clinical relevance of the algorithm, we calculated the percentage of patients correctly classified within 20% of the actual dosage. The percentage of patients correctly classified by the pharmacogenetic algorithm was 46%, whereas 34% were classified properly with the clinical algorithm **(Table 6)**.

**Table 6:** Patients correctly classified (predicted dose within ± 20% of the actual dosage) by genetic and clinical algorithms in the generation, validation and entire cohorts (n = 682).

	% Correctly classified		
	Pharmacogenetic algorithm	Clinical algorithm	p-value
Generation cohort (n = 554)	46.9%	34.7%	< .001
Validation cohort (n = 128)	46.5%	34.1%	< .001
Entire cohort (n = 685)	46.9%	34.5%	< .001

If we classify the patients into the 3 dosage groups (<11 mg/week, 11–21 mg/week, >21 mg/week), we can see that the percentage of patients correctly classified is higher in the intermediate group (11–21 mg/week) for both algorithms, whereas the difference between the 2 algorithms increases in the extreme-dosage groups. Thus, the pharmacogenetic algorithm correctly classified 32.4% of the low-dosage group, compared with 19.9% using the clinical algorithm. Similarly, the pharmacogenetic algorithm correctly classified 41.4% of the high-dosage group compared with only 6.3% with the clinical algorithm. Differences in the MAE were also significant in the extreme-dosage groups between the 2 algorithms **(Table 7).**

**Table 7:** Patients correctly classified (predicted dose within ± 20% of actual dosage) and MAE from the entire cohort (n = 682) by genetic and clinical algorithms according to dosage groups.

Dosage Group	Pharmacogenetic algorithm	Clinical Algorithm	Difference	p-value*
Low (≤11 mg/week)				
% correctly classified	32.4%	19.9%	12.5%	< .001
Mean MAE (SD)	3.12 (2.32)	4.37 (2.78)	1.25 (3.01)	< .001
95% CI			0.85 to 1.66	
Median (11–21 mg/week)				
% correctly classified	58.1%	54.5%	3.6%	.118
Mean MAE (SD)	3.01 (2.32)	3.15 (2.36)	0.14 (2.97)	.403
95% CI			0.18 to 0.45	
High (≥21 mg/week)				
% correctly classified	41.4%	6.3%	34.8%	< .001
Mean MAE (SD)	6.64 (5.26)	10.92 (5.93)	4.28 (3.76)	< .001
95% CI			3.62 to 4.93	

Abbreviations: CI, confidence interval; MAE, mean absolute error (= SQR[(predicted-observed)^2^]); SD, standard deviation.

Published pharmacogenetic algorithms for acenocoumarol show differences in the included clinical/demographic and genetic variables. All account for over 40% of the variability in the dosage of this drug. Table 8 shows the comparisons regarding the performance and included variables between this new algorithm and others.

**Table 8:** Comparison of performance and variables included in the various acenocoumarol algorithms.

			CLINICAL VARIABLES											GENETIC VARIABLES				
Algorithms	R^2^ Derivation cohort	R^2^ Validation cohort	Age	Sex	Weight	Height	Body mass index	Body surface area	Amiodarone use	Enzyme inducer use	Smoking status	Indication for surgery	Target INR	*CYP2C9*	*VKORC1*	*CYP4F2*	*APOE*	*GGCX*
EU-PACT	(n = 375) 52.6%	(n = 168) 49%	X	X	X	X			X					X	X			
LPUH (First algorithm)	(n = 117) 60.6%	(n = 30) 38.8%	X				X		X	X				X	X	X	X	
Cerezo-Manchado	(n = 973) 50%	(n = 2683) 51%	X				X							X	X	X		
Rathore (North Indians)	(n = 125) 41.4%	NA	X	X	X	X		X			X	X		X	X	X		X
Kumar (South Indians)	(n = 217) 61.5%	NA	X				X							X	X	X		X
Our Algorithm	(n = 554) 52.8%	(n = 128) 64%	X	X	X				X	X			X	X	X	X		

Abbreviations: CI, confidence interval; EU-PACT, European Pharmacogenetics of Anticoagulant Therapy; LPUH, La Paz University Hospital; MAE, mean absolute error (= SQR[(predicted-observed)^2^]); NA, not applicable; SD, standard deviation

## Discussion

Expensive new oral anticoagulants with a short history of clinical use are being marketed; however, coumarins such as warfarin, acenocoumarol and phenprocoumon are still the most frequently prescribed drugs for the management of TVD, AF and VR. The high interindividual variability in the dosage requirements of coumarins has been attributed to several clinical and demographic factors and genetic variations. This high variability markedly affects the time within the target INR and the clinical outcomes. Jones et al. showed that a 10% increase in the time out of range was associated with a 29% higher risk of mortality, a 10% increase in the risk of ischemic stroke and a 12% increase in all thromboembolic events [27].

For patients with TVD, the risk of progression, recurrence and death due to PE is greatest in the first weeks after the diagnosis. Despite modern diagnostic and treatment methods, PE continues to have a high mortality rate during the first 3 months, which is probably due to the recurrence of embolism. The frequency of PE could be lowered if more intensive anticoagulation were used [28].

For patients with AF, preventing stroke and thromboembolism are the primary therapeutic goals. For these patients, the expected embolic event rate increases from 2.5% to over 12% per year if they are not properly anticoagulated [29]. Rates of major hemorrhage reported for patients with AF undergoing warfarin treatment range from 1.3 to 7.2 per 100 person-years. Patients with AF tend to be older, have associated comorbidities and take more concomitant medications [29]. The first 90 days are associated with a 3-fold increased risk of major hemorrhage, which is associated with the advanced age of these patients [30]. These data correspond with those of our study; in which patients with AF had the lowest average dose of acenocoumarol, which could increase the likelihood that some patients receive a higher dose of acenocoumarol at the beginning of the treatment. In clinical practice, the estimation of both bleeding and stroke risks are recommended to guide decisions on thromboprophylaxis therapy [31,32].

This study confirms that acenocoumarol algorithms can explain more than 50% of the interindividual variability in the most appropriate dosage of acenocoumarol for stable anticoagulation. The algorithm previously designed and validated by our group was developed for a patient cohort with TVD [23]. Given the fact that these patients are typically younger, have less comorbidity and take fewer concomitant medications, we believe this algorithm might not be useful for other diseases that require anticoagulation with acenocoumarol due to the differences (mainly demographic and clinical) between patients with TVD and those with other diagnoses. Nevertheless, Jimenez-Varo et al., in a recently published study, reported that implementation of the algorithm previously designed by our group led to the highest percentage of correctly classified patients (40.7%) compared with the other algorithms published to date for acenocoumarol [33].

The algorithm described here leads to similar results as those from other algorithms [22,24] performed for the European population regarding the factors influencing stable dosages of coumarins. The most important factors for dosage prediction are the *VKORC1* and *CYP2C9* genotypes, whereas *CYP4F2* has less influence. Regarding clinical variables, age and weight were the most important clinical factors for dosage prediction. Other variables we found significant, such as concomitant medications and target INR, are not consistently included in published algorithms.

When considering the entire cohort, this algorithm explained 55% of the interindividual variability of the most appropriate acenocoumarol dosage; clinical variables explained only 15.1% of this variability. The results show that the inclusion of genetic variables greatly increases the predictability of the dosage required for each patient. Algorithms published for other populations would not be comparable with the results of this algorithm. There are variations in the frequency of polymorphisms among various ethnic groups; some genes have more impact on one ethnic group than another. Thus, in the study performed on the Northern Indian population [20], *VKORC1* and *CYP4F2* polymorphisms were the principal genetic variables, whereas in the algorithm developed for Southern Indians and those for the European population, *VKORC1* and *CYP2C9* are the genetic factors that contribute most to the performance of these algorithms. Furthermore, our algorithm differs from those published for the Indian population [20,21]; the authors of that algorithms included *GGCX* gene polymorphisms (rs11676382), which we do not.

When the predictive performance of the pharmacogenetic algorithm was evaluated by disease, the best prediction was for the AF group (R^2^ of 57.4%), which was similar to the R^2^ found for the VR group. For the patients with TVD, the variability explained for the algorithm was somewhat lower (R^2^ = 51.5%). However, the MAE and %MAE were similar in all groups, confirming that the algorithm worked similarly for all groups.

Not surprisingly, when using standard starting dosages or dosages based on clinical variables, most of the incorrectly dosed patients were those who needed low or high acenocoumarol dosages. These are the patients we can expect to benefit most from the pharmacogenetic algorithm. In this study, the percentage of patients for whom the pharmacogenetic algorithm would have improved acenocoumarol dosing when compared with a clinical algorithm would have been approximately 12.7% (87 patients out a total of 685). Most were patients needing low dosages (27 patients) and notably higher dosages (45 patients). The former would have been overdosed for a number of weeks (until clinical dose adjustments reached a stable INR) and could have been at risk of bleeding, whereas the latter could have been underdosed and would have been at risk of thromboembolic events.

There were differences between the 2 algorithms published by our group regarding the variables included. On one hand, we included the target INR variable in this new algorithm, as justified by the different target INRs for the various diagnoses. We found that the target INR had a modest but significant influence (see Table 3). On the other hand, the contribution of *APOE* to the total variability was not confirmed in this study because it was not significant (p = 0.521, R^2^ = 0.1%), and was therefore not included in the algorithm. The inclusion in the algorithm of the taking of certain drugs such as enzyme inducers and amiodarone, is also justified by data in the literature on their influence on the coumarin dose and, as we have seen in the model, their influence is significant (beta of 0.070 and -0.337 respectively). Amiodarone intake was one of the variables included in the algorithm published by the EU-PACT group, with a similar result (beta of -0.377).

The usefulness of pharmacogenetic algorithms in a clinical setting has been tested in 3 published clinical trials, 2 of which were performed with warfarin and 1 with acenocumarol and phenprocoumon. In 2013, a randomized trial of genotype-guided acenocoumarol dosing showed that the pharmacogenetic algorithm, which included clinical and genetic variables, did not improve the percentage of time in the therapeutic INR range during the 12 weeks after the start of therapy compared with the clinical algorithm [34]. The EU-PACT group, however, reported that pharmacogenetics-based warfarin dosing was better than standard dosing, with a higher percentage of time in the therapeutic INR range than traditional clinical dosages during the start of warfarin therapy [35]. The results of these studies are controversial in terms of the applicability of pharmacogenetic algorithms, and certainly there are variables that are not included in these algorithms, which in the context of clinical trials cannot be controlled.

Pharmacogenetic algorithms have typically been developed by collecting information from individuals with various demographic, clinical and genetic characteristics. Both clinical and pharmacogenetic algorithms show similar accuracy in predicting the dose of wild-type genotype patients; however, algorithms that integrate genetic information are more accurate and useful for patients with allelic variants because these patients often have extreme dosages of acenocoumarol, for whom INR stabilization becomes more difficult and time-consuming to achieve.

There are a number of limitations in this study. A number of parameters that have also been linked to changes in the required stable coumarins dosages were not included, such as smoking status, other concomitant medications and the dietary vitamin K intake. All are important factors to keep in mind when establishing a stabilized dosage of acenocoumarol.

In conclusion, our new algorithm can provide better prediction, when compared with the clinical algorithm, of the ideal acenocoumarol dosage for a broad spectrum of patients who are starting treatment with this drug, particularly those who need extreme dosages. The performance of the pharmacogenetic algorithm is similar for patients with different diseases despite the demographic and clinical variations among the patients.

## Supporting Information

S1 Appendix PGx-ACE Investigation Group. (DOCX)
