# Supplementary material for: A New Pharmacogenetic Algorithm to Predict the Most Appropriate Dosage of Acenocoumarol for Stable Anticoagulation in a Mixed Spanish Population
Source: PLoS One. 2016 Mar 15;11(3):e0150456. doi: 10.1371/journal.pone.0150456 (PMC4792430; doi:10.1371/journal.pone.0150456)
Supplement: S1 Appendix — (DOCX) [file pone.0150456.s001.docx]

**S1 Appendix**:

**PGx-ACE Investigation Group:**

Claudia Zegarra^1^, Marta Velasco^1^, Mario Muñoz^1^, Rafael Hernández^1^, Elena Ramírez^1^, Jesús Frías^1^, Alicia Lorenzo^2^, María Ángeles Rodríguez Dávila^2^, Giorgina Salgueiro^2^, María V. Cuesta-García^3^, M. Soledad García-Muñoz^3^, María Teresa Gómez Rodríguez^4^, Pilar Pérez Egea^4^, Carlos Casanova García^4^, Laura García Regaño^4^, María Azucena Saez Berlana^4^, María Alejandra Rabanal Carrera^4^, María Pilar Martin Cerrato^4^, Manuel Sánchez López^4^, Pablo Astorga Díaz^4^, Milagros Velázquez García^4^, Francisco Javier De La Casa Sánchez^4^, Ángeles Conde Llorente^4^, María Dolores Parejo De Pablos^4^, María Patrocinio Verde González^4^, María Rosa Del Álamo Gutiérrez^4^, Ángeles Brieva García^4^, Margarita Encinas Sotillo^4^, Esther Frías Díaz^4^, Francisca Dacal Cubillo^4^, Inés Casas Jiménez^4^, Isabel Sola Vergara^4^, Fernando López Beltrán De Lis^4^, Antonio Vázquez Gallego^4^, María Teresa García Argudo^4^, María Ángeles Bueno Martin^4^, Encarnación Vega Arija^4^, Carmen Belinchón Moya^4^, Gloria Menal Arriazu^4^, María Teresa Gómez Martínez^4^, Ana Román Ruiz^4^, Laura Martin Saez^4^.

1. Department of Clinical Pharmacology. La Paz University Hospital, IdiPAZ, Madrid, Spain
2. Department of Internal Medicine. La Paz University Hospital, IdiPAZ, Madrid, Spain
3. Department of Hematology. La Paz University Hospital, Madrid, Spain
4. Primary Health Care Center “Barrio del Pilar”, Madrid, Spain
